# Supplementary material for: Identification of photoperiod-induced specific miRNAs in the adrenal glands of Sunite sheep (Ovis aries)
Source: Front Vet Sci. 2022 Jul 22;9:888207. doi: 10.3389/fvets.2022.888207 (PMC9354845; doi:10.3389/fvets.2022.888207)
Supplement: Supplementary file 1 [file Data_Sheet_1.ZIP › Supplementary file/miRDeep2 file.zip!/miRDeep2 file/Novel_Structure/LP42A2/Novel_8.pdf]

Provisional ID	:	NC_019476.2_29856
Score total	:	1
Score for star read(s)	:	-1.3
Score for read counts	:	0
Score for mfe	:	0.7
Score for randfold	:	1.6
Score for cons. seed	:	
Total read count	:	203
Mature read count	:	202
Loop read count	:	0
Star read count	:	1

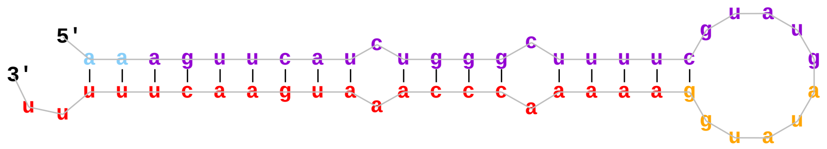

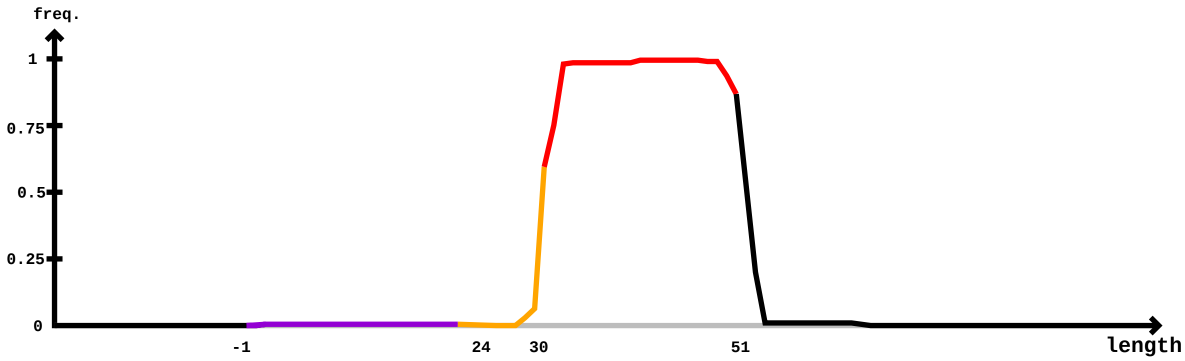


```
                 Star                         Mature
5'- uaauacuggauugaccaaaaaguucaucugggcuuuucguaugauauggaaaaacccaaaugaacuuuuugaccaacccaauaccuauuuguaugcuucuccugcucaguugc   -3'   obs
    uaauacuggauugaccaaaaaguucaucugggcuuuucguaugauauggaaaaacccaaaugaacuuuuugaccaacccaauaccuauuuguaugcuucuccugcucaguugc         exp
    ......(((.(((..((((((((((((.((((.(((((..........))))).)))).))))))))))))..))).)))((((......))))((.......))........    reads    mm     sample
    ....................aguucaucugggcuuuucguaug......................................................................    1        0      seq
    ...............................................ggaaaaacccGaaugaacuuu.............................................    1        1      seq
    ...............................................Ugaaaaacccaaaugaacuuu.............................................    2        1      seq
    ...............................................ggaGaaacccaaaugaacuuu.............................................    1        1      seq
    ...............................................Ugaaaaacccaaaugaacuuuu............................................    1        1      seq
    ...............................................ggaaaaacccaaaugaacuuuA............................................    1        1      seq
    ................................................gaaaaacccaGaugaacuuuu............................................    6        1      seq
    ................................................gaaaaacccaGaugaacuuuuu...........................................    1        1      seq
    .................................................aaaaacccaGaugaacu...............................................    1        1      seq
    .................................................aaaaacccaGaugaacuuu.............................................    7        1      seq
    .................................................aaaaacccGaaugaacuuuu............................................    2        1      seq
    .................................................aaaaacccaGaugaacuuuu............................................    4        1      seq
    .................................................Caaaacccaaaugaacuuuuu...........................................    1        1      seq
    .................................................aaaaGcccaaaugaacuuuuu...........................................    2        1      seq
    .................................................aaaaCcccaaaugaacuuuuu...........................................    2        1      seq
    .................................................aaaaacccaaaugaacuuuuG...........................................    1        1      seq
    .................................................aaaaacUcaaaugaacuuuuu...........................................    1        1      seq
    .................................................aaaaacccaaaugaacuuuuA...........................................    2        1      seq
    .................................................aaaaacccaGaugaacuuuuu...........................................    42       1      seq
    .................................................aaaaacccaaaugaacuuuuuA..........................................    6        1      seq
    .................................................aaaaacccaaGugaacuuuuug..........................................    1        1      seq
    .................................................Caaaacccaaaugaacuuuuug..........................................    3        1      seq
    .................................................aaaaacccaaaugaacuuuuuU..........................................    2        1      seq
    .................................................aaaaacUcaaaugaacuuuuug..........................................    9        1      seq
    .................................................aaaaacccaGaugaacuuuuug..........................................    2        1      seq
    .................................................aaaaGcccaaaugaacuuuuug..........................................    6        1      seq
    .................................................aaaGacccaaaugaacuuuuuga.........................................    2        1      seq
    .................................................aaaaacccaaaugaacuuuuugU.........................................    5        1      seq
    .................................................aaaaacccaaaugaacuuuuuga.........................................    4        0      seq
    .................................................aaaCacccaaaugaacuuuuuga.........................................    1        1      seq
    .................................................aaaaGcccaaaugaacuuuuuga.........................................    3        1      seq
    ..................................................aaaacccGaaugaacuuuuu...........................................    1        1      seq
    ..................................................aaaacccaaaugaacuuuuug..........................................    12       0      seq
```

Star **Mature**

```
uaauacuggauugaccaaaaaguucaucugggcuuuucguaugauauggaaaaacccaaaugaacuuuuugaccaacccaauaccuauuuguaugcuucuccugcucaguugc
..................................................aaaacccaaGugaacuuuuug..........................................    1    1    seq
..................................................aaaacUcaaaugaacuuuuug..........................................    6    1    seq
..................................................aaaacccaaaugaacuuuuuU..........................................    1    1    seq
..................................................aCaacccaaaugaacuuuuug..........................................    1    1    seq
..................................................aaaacccaaaugaacuuuuuA..........................................    4    1    seq
..................................................aaaacccaaaugaacuuuuugU.........................................    4    1    seq
..................................................aaGacccaaaugaacuuuuuga.........................................    2    1    seq
...................................................aaacccGaaugaacuuuuu...........................................    28   1    seq
...................................................aaacccaaaugaacuuuuug..........................................    2    0    seq
...................................................aaacccaaaugaacuuuuugU.........................................    1    1    seq
...................................................aaacccGaaugaacuuuuuga.........................................    16   1    seq
....................................................aacccaaaugaacuuuuuga.........................................    1    0    seq
...........................................................augaacuuuuugaccaacccaaA...............................    1    1    seq
...........................................................augaacuuuuugaccaacccaaAa..............................    1    1    seq
```
